# Supplementary material for: Critical Role of Etching Parameters in the Evolution of Nano Micro SLA Surface on the Ti6Al4V Alloy Dental Implants
Source: Materials (Basel). 2021 Oct 23;14(21):6344. doi: 10.3390/ma14216344 (PMC8585160; doi:10.3390/ma14216344)
Supplement: Supplementary file 1 [file materials-14-06344-s001.zip › materials-1380662-supplementary.pdf]

Supplimentry File

Critical Role of Etching Parameters in the Evolution of Nano Micro SLA Surface on the Ti6Al4V Alloy Dental Implants

Pankaj Chauhan [1,2], Veena Koul [2] and Naresh Bhatnagar [1,*]

[1] Mechanical Engineering Department, Indian Institute of Technology Delhi, India, 110016; dr.prachichauhan@gmail.com
[2] Centre for Biomedical Engineering, Indian Institute of Technology Delhi, India, 110016; veenak_iitd@yahoo.com
* Correspondence: nareshb@mech.iitd.ac.in

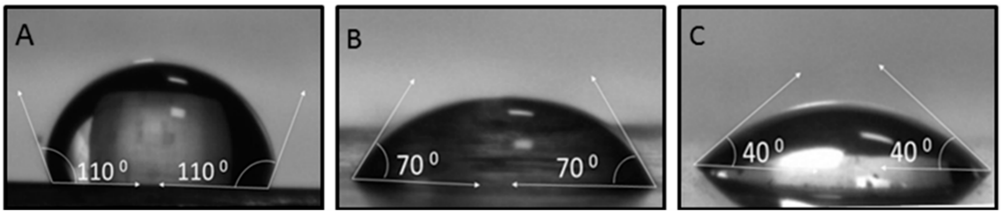

Fig. S1: Contact angle measurement on A) Machined, B) Acid-etched at RT (10 min), C) Acid-etched at HT (5 min.)

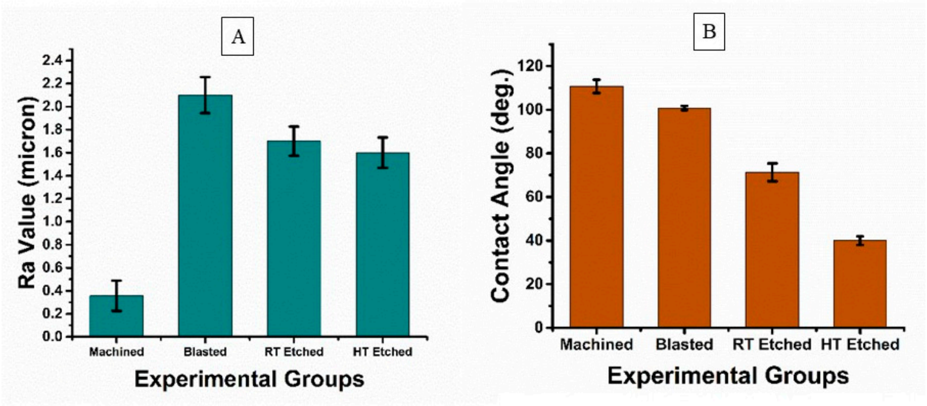

Fig. S2: Bar Diagram showing A) Ra values and B) contact angle on machined and experimental surfaces

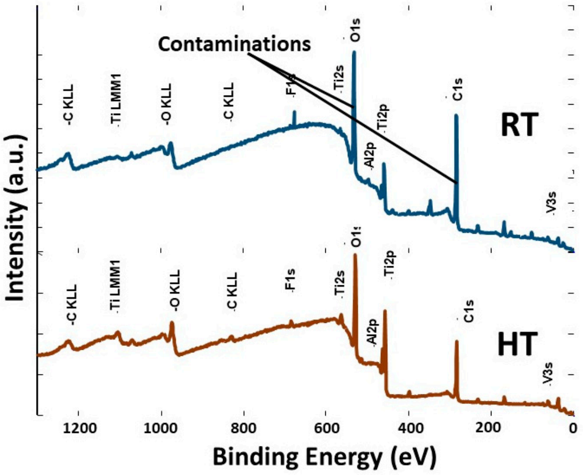

Fig: S3: XPS spectra of RT and HT etched surfaces

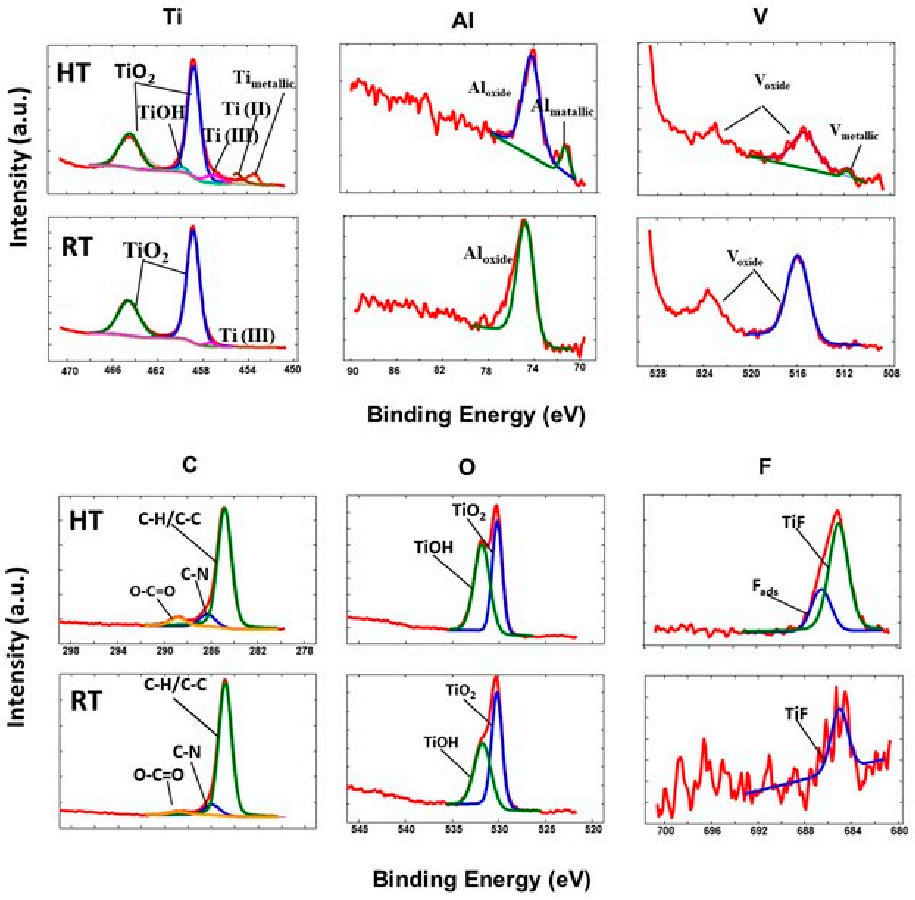

Fig. S4: High resolution deconvoluted XPS spectra of core level Ti2p, Al2p, V2p, C1s, O1s and F1s at the surface of Titanium surface etched at room temperature and high temperature
